# Targeting estrogen to the brain via DHED does not seem to protect against metabolic dysfunction in obese OVX mice

**DOI:** 10.1210/endocr/bqag082

**Published:** 2026-09-01

**Authors:** Celine Camon, Elodie Kip, Rebecca Lord, Caroline Decourt, Mel Prescott, Jenny Clarkson, Norma P Sandoval, Paul B Vander, J Edward Van Veen, Katalin Prokai-Tatrai, Stephanie M Correa, Rebecca E Campbell, Michael Garratt

**Affiliations:** Centre for Neuroendocrinology, University of Otago, Dunedin 9016, New Zealand; Department of Integrative Biology and Physiology, University of California Los Angeles, Los Angeles, CA 90095, USA; Centre for Neuroendocrinology, University of Otago, Dunedin 9016, New Zealand; Centre for Neuroendocrinology, University of Otago, Dunedin 9016, New Zealand; Centre for Neuroendocrinology, University of Otago, Dunedin 9016, New Zealand; Centre for Neuroendocrinology, University of Otago, Dunedin 9016, New Zealand; Centre for Neuroendocrinology, University of Otago, Dunedin 9016, New Zealand; Department of Integrative Biology and Physiology, University of California Los Angeles, Los Angeles, CA 90095, USA; Department of Integrative Biology and Physiology, University of California Los Angeles, Los Angeles, CA 90095, USA; Department of Integrative Biology and Physiology, University of California Los Angeles, Los Angeles, CA 90095, USA; Department of Pharmacology and Neuroscience, University of North Texas Health Science Center, Fort Worth, TX 76107, USA; Department of Integrative Biology and Physiology, University of California Los Angeles, Los Angeles, CA 90095, USA; Centre for Neuroendocrinology, University of Otago, Dunedin 9016, New Zealand; Centre for Neuroendocrinology, University of Otago, Dunedin 9016, New Zealand

**Keywords:** DHED, estrogen, menopause, metabolic health, hypothalamus

## Abstract

Menopausal hormone therapy (MHT), prescribed for climacteric symptoms including hot flushes and weight gain, contains estrogens such as 17 beta-estradiol (17βE2). However, estrogen receptor activation by MHT may increase reproductive cancers in some people. As the protective metabolic effects of 17βE2 are partly mediated through the arcuate nucleus (ARC) of the hypothalamus, restricting 17βE2 actions to the brain could serve as a safer method for MHT. The prodrug 10β,17β-dihydroxyestra-1,4-dien-3-one (DHED) is enzymatically converted into 17βE2 exclusively within the brain. DHED has already demonstrated positive benefit in rodent models of hot flushes and cognitive decline, while avoiding peripheral hormonal burden. Therefore, we hypothesized that DHED treatment in obese female mice would act within the hypothalamus to provide the same beneficial metabolic effects as 17βE2. Female mice were ovariectomized, placed on a high fat diet and split into control, 17βE2, or DHED treatment groups. Body weight, uterine weight, glucose tolerance and peripheral concentrations of luteinizing hormone were recorded, along with the expression of the progesterone receptor and kisspeptin mRNA within the ARC. Delivery of DHED at a similar dose as 17βE2 failed to improve metabolic parameters or recapitulate the hypothalamic responses induced by 17βE2. Our findings subsequently show that delivery of DHED at higher doses elicited estrogen-like actions within the brain but still failed to improve metabolic health. Additional investigations surrounding DHED's conversion to 17βE2 within the hypothalamus are warranted to conclude whether estradiol actions exclusively within the central nervous system can independently regulate protective effects on metabolism.

The loss of follicular reserve, reduction of circulating ovarian hormones, and the eventual cessation of menstruation mark the transition to menopause (1). Hormonal changes that occur over this period include the relatively abrupt decline of estrogen and progesterone, with the simultaneous reduction in inhibin and anti-Müllerian hormone and an increase in follicle-stimulating hormone (2). Many people experience symptoms during this transition that can significantly alter their quality of life, including hot flushes, significant weight gain, sleep disturbances, and mood alterations, which are thought to be driven (either directly or indirectly) by estrogen deprivation to the brain (3, 4).

A shift occurs in metabolic function during menopause, which leads to adipose tissue redistribution from femoral and gluteal subcutaneous deposits and results in increased visceral adiposity (5). Other markers of poor metabolic health observed during and after menopause include elevated cholesterol, impaired glucose tolerance, and increased inflammatory markers (6, 7). These alterations can increase insulin resistance, lipolysis and elevate the overall risk for metabolic disease (8, 9). The dramatic decline of ovarian 17 beta-estradiol (17βE2) production is thought to play a significant role in the redistribution of adiposity and associated metabolic disturbances during this period (10). This is mirrored in ovariectomized (OVX) rodents that demonstrate increased abdominal fat, increased body weight and adiposity, and impaired glucose tolerance (11-15). When OVX mice on a high fat diet (HFD) are treated with 17βE2, they show significantly reduced body weight, adipocyte size, food intake, hepatic triglyceride levels, and a marked improvement in glucose tolerance, highlighting the therapeutic potential of estrogen replacement in managing metabolic health in menopause (16, 17). While the safety profile of menopausal hormone therapy (MHT) has been well characterized over the last decade, MHT can be unsuitable for people with, or histories of, reproductive cancers (18). Increased cancer risk stems from undesirable hormone-receptor signaling actions within estrogen-sensitive tissues in the periphery, including the uterus and breast (19). Therefore, alternatives to traditional hormonal treatments are required to provide safe and effective treatments to alleviate menopausal symptoms.

The specific tissues where 17βE2 acts to improve metabolic health are not fully established. However, the hypothalamus, and the arcuate nucleus (ARC) in particular, is likely to play a major role due to its importance in the regulation of body weight, metabolism and food intake (20-22). Estrogen receptor alpha (ERα), the canonical estrogen receptor activated by 17βE2, is highly expressed in the ARC (23-25). Ablation of neuronal ERα induces abdominal obesity and hyperphagia in female mice and ERα deletion exclusively from anorexigenic pro-opiomelanocortin *(*POMC*)* neurons also induces hyperphagia (21). Neurons in the ARC are responsible for sensing circulating levels of leptin, insulin, and glucose (26). Neurons expressing ERα in the neighboring ventromedial hypothalamus of the hypothalamus have also been shown to sense glucose levels and prevent hypoglycemia in female mice, along with playing a key role in thermoregulation (27, 28). Given that the ventromedial hypothalamus projects to the ARC, it is likely that metabolic function is also modulated at least partially by this brain region (29).

10β,17β-dihydroxyestra-1,4-dien-3-one (DHED) is a unique prodrug of 17βE2 that selectively produces the hormone in the brain to treat neurological conditions in rodent models of hot flush, stroke, cognitive decline, and depression (30-33). Consequently, administration of DHED does not increase uterine weight, and estrogen response element (ERE) reporter mice indicate that DHED does not activate ERα in peripheral tissues (30, 33). The conversion of DHED to 17βE2 occurs via a central nervous system–specific enzyme, a short-chain NADPH-dependent dehydrogenase/reductase that is expressed exclusively within and abundantly across the brain, including within the hypothalamus (30). Therefore, DHED may be a novel method of delivering 17βE2 to hypothalamic centers involved in mediating metabolic health, without acting on peripheral tissues.

We hypothesized that DHED treatment would recapitulate the protective metabolic effects of 17βE2 in obese OVX female mice, while bypassing the activation of peripheral estrogen-sensitive organs. We assessed whether DHED treatment could improve a range of metabolic health parameters, including body weight, food intake, glucose tolerance, and liver adiposity in OVX mice on a HFD, and how these responses compared to mice who underwent 17βE2 treatment. ERα- dependent central effects were assessed by measuring progesterone receptor (PR) and kisspeptin expression in the ARC, both of which are sensitive to estradiol signaling, along with peripheral levels of luteinizing hormone (LH) (31, 34, 35).

Previous proof-of-concept studies investigating the utility of DHED-derived 17βE2 to provide protection against cognitive decline, hot flushes, and depression have used similar doses of DHED to 17βE2 (30, 31). We therefore first tested whether DHED treatment at the same dose as 17βE2 would recapitulate estrogen-like actions within the hypothalamus and on metabolic health. We then performed an acute 48-hour dose-response study to identify a dose of DHED with sufficient activation within the brain. We subsequently performed another chronic study with increasing doses of DHED on a HFD to investigate whether optimized dosages of this brain selective prodrug of the hormone could provide protection against metabolic dysfunction.

## Methods

### Animal husbandry

All research was approved by the University of Otago Animal Ethics Committee (AUP-22-42) (Experiments 1 and 3) or by the UCLA Institutional Animal Care and Use Committee (IACUC) (Experiment 2). All animals were of the C57BL/6J strain, sourced from either the Biomedical Research Facility (University of Otago, experiments 1 and 3), or bred in-house or sourced directly from Jackson Laboratories (UCLA, experiment 2). Mice were housed in a temperature- (21 °C) and light-controlled environment on a 12:12 light:dark cycle and had ad libitum access to food and water.

### Experimental timelines

#### Experiment 1: metabolic function and hypothalamic response with a matched dosage of DHED and 17βE2.

Female C57BL/6J female mice were ovariectomized (OVX, n = 20) between 6 and 8 weeks of age (Fig. 1a). Prior to OVX, mice were fed a normal chow diet (Teklad Global 18% Protein Rodent Diet #2918 (protein: 18.6%; fat 6.2%; carbohydrate: 44.2%). Mice were then placed on a 45% HFD (SF04-001, 35.8% CHO, 18.5% PRO, 45% FAT) from Specialty Feeds (Glen Forrest, WA) for 8 weeks before being randomly assigned to 1 of 3 different hormone treatment groups. Animals remained on HFD for another 25 days until the end of the experiment. Ovariectomy surgeries were conducted under 2% isoflurane and mice received Carprofen (0.074 mg/kg) and buprenorphine (0.1 mg/kg) at the time of the surgery, with both drugs administered again at the same dose 24 hours after surgery. For hormone treatment, mice were implanted with osmotic mini-pumps (as outlined below) that delivered vehicle (propylene glycol, n = 6), 17βE2 (5 μg/day, n = 7), or DHED (5 μg/day, n = 7) treatment for 25 days. We conducted a power analysis to determine the sample size required to detect an effect of 17βE2 treatment on body weight, which we considered the primary variable and a composite factor that reflects a change in energy balance with treatment. Previous studies have reported strong effects of 17βE2 treatment on body weight changes after 4 weeks in the context of HFD treatment (16, 17, 36) and power analysis (power at 0.8 and significance at α = 0.05) using the mean body weight change and pooled SD from previous studies indicated a small sample size (3-4 per group) was required to detect a similar magnitude change (16). We allowed 7 animals per group (6 in controls) to account for any loss of animals over the experiment and to detect any weaker effects that may occur with other outcomes or DHED treatment. Mice were weighed weekly while on the HFD, and then every second day while receiving hormone treatment. Body weight and food intake were measured from 4 days postsurgery so that any food or weight changes were not a consequence of acute adverse responses to surgery. Glucose tolerance tests (GTTs) were performed after 23 days of pump insertion and mice were euthanized via transcardial perfusion with 4% paraformaldehyde (PFA) for tissue collection after a total of 25 days of pump insertion.

**Figure 1:**
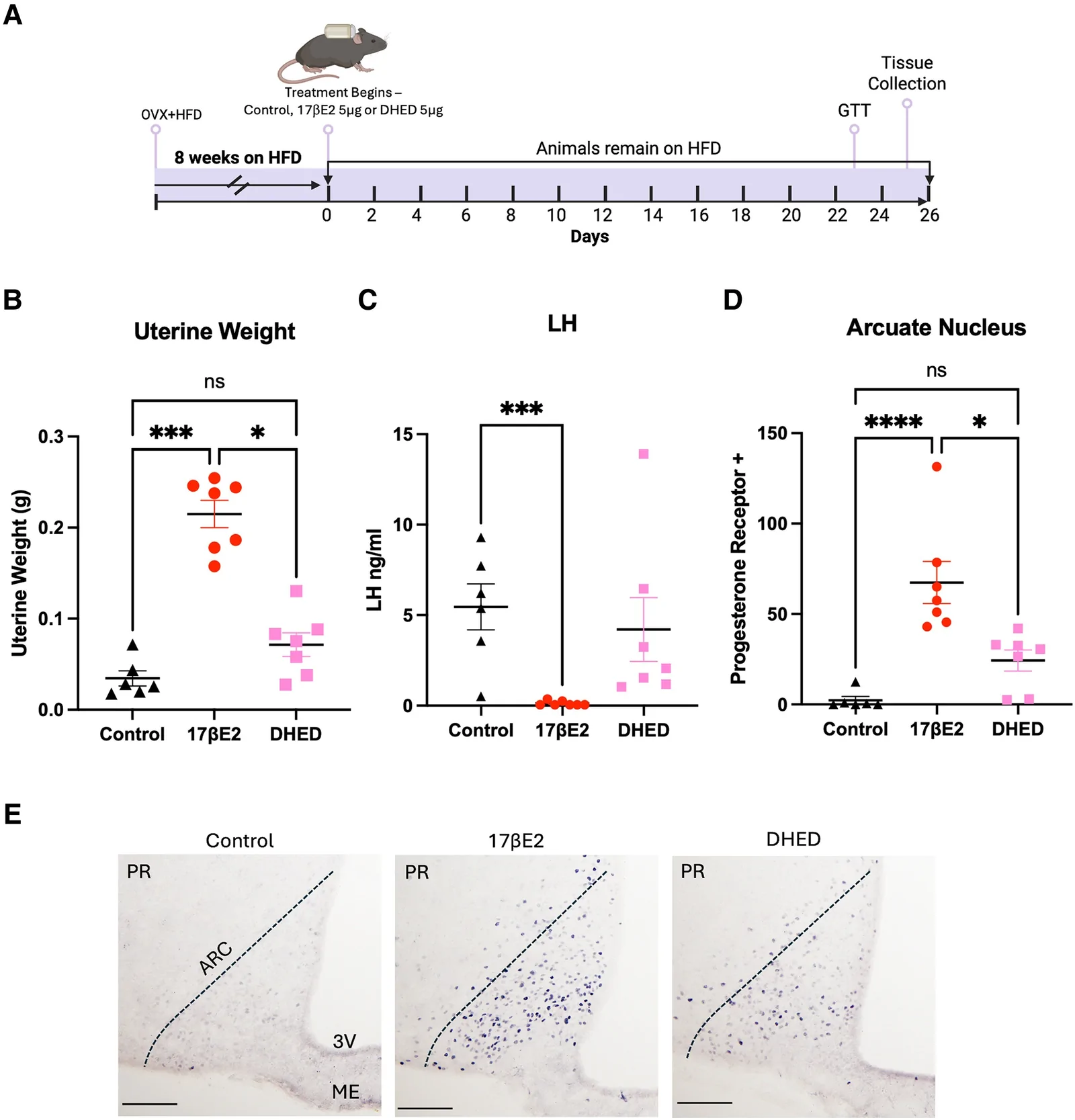
Experiment 1. DHED does not increase uterine weight and influences progesterone receptor expression in the ARC in some animals. a, timeline: Animals were ovariectomized and placed on a 45% HFD for 8 weeks, then osmotic mini-pumps delivered treatment for 25 days. n = 6 for vehicle treatment, n = 7 for 5 μg/day 17βE2 treatment, n = 7 for 5 μg/day DHED treatment. b, Uterine weight for each animal. c, LH levels for each animal. d, Progesterone receptor (PR) abundance in the ARC. e. Representative images of PR abundance: vehicle control, 17βE2 or DHED treatment. *P < 0.05, ***P = 0.001, ****P < 0.0001, (Dunn's post hoc test). Scale bar = 100 µm. Error bars show mean ± SEM. Abbreviations: ARC, arcuate nucleus; 3V, third ventricle; ME, median eminence.

#### Experiment 2: Acute DHED dose-response study to assess central actions.

C57BL/6J mice maintained on a normal chow diet (Lab Diet, Mouse Diet 9F, (protein: 23%; fat 22%; carbohydrates: 55%) underwent OVX at 7 to 9 weeks of age and were allowed to recover for 1 week before being randomly split into groups that received either control (n = 6, sesame oil), 17βE2 (5 μg/day, n = 4), or 1 of 3 increasing doses of DHED (5 μg/day [n = 5], 10 μg/day [n = 4], 20 μg/day [n = 5]). The effect of estrogen treatment on PR expression is very strong, with all treated animals typically having high levels compared to OVX animals (ie, Fig. 1d) (37). Power analysis indicated a minimum sample size for statistical analysis is required (ie, n = 3 per group) to detect an effect of 17βE2 treatment on PR expression; we therefore allowed 4 to 6 animals per group to account for any loss during the experiment or additional variability within DHED-treated animals. DHED or 17βE2 was dissolved in 3% ethanol in sesame oil and administered once daily for 2 days via a subcutaneous injection. On day 2, animals were euthanized via transcardial perfusion with 4% PFA 6 hours after injection, a timeframe that is sufficient to observe increases in PR expression in the hypothalamus following 17βE2 treatment (38).

#### Experiment 3: Prolonged DHED dose-response study to assess metabolic effects

C57BL/6J mice were ovariectomized (n = 40) between 7 and 10 weeks of age. Prior to OVX, mice were fed a normal chow diet (Teklad Global 18% Protein Rodent Diet #2918 (protein: 18.6%; fat 6.2%; carbohydrate: 44.2%). Immediately following OVX, mice were placed on a 45% HFD (TestDiet 58V8, 35.8% CHO, 18.1% PRO, 46.1% FAT, semi-purified) from TestDiet (Richmond, IN) for 4 weeks. Animals were then randomly assigned to 1 of 5 different treatment groups and remained on a HFD while undergoing treatment for the remainder of the experiment. Mice then underwent surgery to insert osmotic mini-pumps that delivered vehicle (polyethylene glycol, n = 7), 17βE2 (5 μg/day, n = 6), or 1 of 3 increasing doses of DHED (5 μg [n = 6 per group], 10 μg [n = 6 per group], 20 μg, [n = 7 per group]) for 14 days. Mice were weighed weekly while on a HFD, and then every second day along with food intake while receiving hormone treatment. Body weight and food intake were measured for the first 10 days. Glucose tolerance tests (GTTs) were performed on day 13 and mice were euthanized via transcardial perfusion the following day.

### Metabolic analyses

Mice were weighed weekly while on a HFD, and then every second day while osmotic mini-pumps delivered treatments in experiments 1 and 3. Food intake was assessed for pairs of mice in experiments 1 and 3, with the average daily food intake value per cage used for analysis.

After mice had been on hormone treatment for 23 days (experiment 1) or 13 days (experiment 3), mice were fasted for 6 hours at 7 Am to 8 Am at the beginning of the light period (water access remained ad libitum) prior to intraperitoneal (ip) injection with 2 g/kg D-glucose (n = 6-7 per group). Blood glucose levels were measured with a glucose meter (Accu-CHEK, Roche) immediately pre-injection (time 0) and at 15, 30, 45, 60, 90, and 120 minutes postinjection. For mice whose levels surpassed the Accu-CHEK meter range, the highest glucose reading possible of 33.3 mmol/L was used for that time point.

### Hormone administration via osmotic mini-pumps

For experiments 1 and 3, vehicle, 17βE2 and DHED treatments were delivered by subcutaneous infusion via osmotic mini-pumps (Alzet, CA, USA, Model 2004, delivery rate 0.25 ± 0.05 μL/h for 25 days (experiment 1), Alzet, CA, USA, Model 1002, delivery rate 0.25 ± 0.05 μL/h for 14 days (experiment 3). In experiment 1, 17βE2, DHED and vehicle solutions were dissolved and diluted in 100% propylene glycol so as to deliver 5 μg of either hormone per day. In experiment 3, 17βE2, DHED, and vehicle solutions were dissolved in 50% DMSO in polyethylene glycol 300 so as to dispense the required daily dosages. Prefilled pumps were activated prior to insertion in a sterile saline solution (0.9%) in a 37 °C water bath for 48 hours (experiment 1) or overnight (experiment 3). Mini-pump insertion took place while animals were anesthetized under 2.5% to 3% isoflurane. Carprofen (0.074 mg/kg) and buprenorphine (0.1 mg/kg) were administered at the time of the surgery, and both were administered again 24 hours after surgery.

### Euthanasia/tissue collection

At the end of each experiment mice were anesthetized with pentobarbital (150 mg/kg) and upon loss of the pedal reflex, the uterus and a segment of liver were dissected. The animal was then perfused transcardially with 4% PFA in phosphate-buffered saline (PBS). Brains were dissected, post-fixed for 24 hours in 4% PFA in PBS, then immersed in 30% sucrose in PBS for another 24 hours. Brains were then frozen using dry ice and ethanol, embedded in optimal cutting temperature compound (OCT) and stored at −80 °C.

### Experiments 1 and 3: immunohistochemistry for chromogenic labeling of PR and fluorescent labeling of Iba1

Brains were coronally sectioned in a series of 4 at 30 microns using a cryostat and stored in cryoprotectant at −20 °C prior to processing. From each animal, 2 to 3 medial ARC sections were anatomically matched and were treated with 30% hydrogen peroxide solution in methanol and Tris Buffer Saline (TBS) prior to blocking (5% normal goat serum [NGS], TBS, 0.25% bovine serum albumin [BSA]). Sections were incubated at 4 °C in a custom primary antibody against PR (1:2500, RC269, AB_2924988) for 60 hours (39, 40). Sections were then incubated for 60 minutes at room temperature (RT) in a secondary biotinylated immunoglobulin antibody (goat anti-rabbit, 1:250, Vector Laboratories Cat# BA-1000, RRID: AB_2313606) and then in Vectastain Elite Avidin-Biotin dissolved in antibody dilution solution (5 mL/mL solution, Vector Laboratories) for 90 minutes at RT. Benzene derivative DAB (3,3′-diaminobenzidine), nickel, and glucose oxidase were used as a substrate to reveal a purple/brown derivative stain within the nucleus of positively stained cells for PR (n = 6-7 per group). Sections were then washed, mounted, dried, and dehydrated through a series of increasing concentrations of ethanol (50%, 70%, 90%, 100%) followed by 100% xylene and cover-slipped using dibutylphthalate plasticizer xylene (DPX) mounting medium (Merck, #100579).

The number of PR-positive cells in the ARC were quantified in 2 to 3 middle ARC sections (Paxinos coordinates: −1.46 to −1.70) from each mouse and averaged across sections. Sections were imaged on an Olympus 1501 microscope with a 10× objective. PR-positive cell counts were automated with ImageJ software (experiment 1) or Qu Path (experiment 3) with constant thresholding throughout the analysis. The investigator was blinded to the treatment group and conducted thresholding based on visible positive staining in the ARC. Briefly, the ImageJ protocol was as follows: The standardized region of interest was outlined in each image (pixel size 0.2406 µm, 10× objective), the background was subtracted, and the particle analysis was conducted with the following measurements: rolling ball radius set to 250, pixel size 100-infinity, circularity 0.25-1. Only PR-positive cells with a stained nucleus were included in counts. Qu Path analysis was performed with optical density sum; nucleus detection parameters were sigma 1.5 µm, background radius 8 µm, and area range 15 to 60 µm^2^; intensity thresholds were 0.2 to 0.25 with maximum background intensity 2 and split by shape enabled; cell expansion was set to 2 µm including the nucleus; and smooth boundaries with measurement recording were applied.

For microglial staining, ARC sections were incubated in blocking solution (5% NGS) for 1 hour at RT, followed by incubation with rabbit anti-Iba-1 (microglia/macrophage calcium-binding protein) primary antibody (1:1000; Fujifilm Wako, RRID: AB_839504) in incubation buffer containing 2% NGS for 48 hours at 4 °C. Sections were then incubated with Alexa Fluor 488 goat anti-rabbit secondary antibody (1:500; Invitrogen, RRID: AB_143165) for 3 hours at RT. Sections were mounted onto gelatin-coated slides and cover-slipped with Fluoromount.

Immunolabeled sections were imaged using a Nikon A1 confocal microscope with a 10× objective and 1.5× digital zoom (pinhole = 4). Unilateral middle ARC Images (1/mouse) were acquired. Images for each experiment were acquired on the same day using identical acquisition settings to minimize variability between sessions (HV: 81 for experiment 1 and 87 for experiment 3; offset: −3; laser intensity (488): 4.33). Microglial coverage was quantified in Fiji (ImageJ) as the percentage of ARC area occupied by Iba-1 immunoreactivity. The same ARC-shaped region was analyzed across sections using a consistent threshold range, with adjustments applied when images were oversaturated.

### Experiment 2: immunohistofluorescent labeling of PR

From each animal. 2 to 3 middle ARC sections were anatomically matched and mounted onto super frost slides (n = 4-5 per group). Slides were washed in PBS then blocked in a solution containing 10% NGS in 0.3% PBS-Triton for 1 hour, then incubated in a primary antibody against PR (1:1000, Abcam Cat# ab101688, RRID: AB_10715248) overnight in a dehumidified chamber. Sections were washed in PBS and then incubated for 2 hours at RT in an AlexaFluor647 conjugated secondary antibody (goat anti-rabbit, 1:1000, Invitrogen, Cat# A-11035, RRID: AB_2534093). Sections were washed once more in 1× PBS before being counterstained with nuclei marker (DAPI, 1:500) and cover-slipped using Prolong Gold Antifade liquid mountant (Southern Biotech, 0100-01).

Images that captured both sides of the ARC were collected using an Olympus 1501 microscope (20×), but unilateral (one side) counts were used for analysis. The average unilateral count from 2 to 3 middle ARC sections from each mouse were used. PR-positive cells were counted manually using the cell counter function in FIJI software. The investigator was blinded to treatment group. The ARC boundaries were defined consistently throughout the analysis. Only cells with PR-immunoreactive nucleus and a positive stain for the DAPI nuclear marker were included in counts.

### *Kiss1* RNAscope

Tissue underwent processing according to the manufacturer's instructions for the RNAscope Multiplex Fluorescent Detection Kit V2 (Advanced Cell Diagnostics, CA, USA). ARC containing sections (2-3/mouse, Paxinos and Franklin mouse brain atlas mouse brain coordinates from −1.46 to 1.70 from Bregma) were washed in 1× diethylpyrocarbonate (DEPC)-treated PBS, baked at 60 °C for 30 minutes then fixed in prechilled neutral buffered formalin (NBF) at 4 °C. Tissue was then dehydrated using several dilutions of ethanol (50%, 70%, 95%, and 100%), rinsed with DEPC-treated PBS and allowed to air dry for 20 minutes. Sections were treated with hydrogen peroxide for 10 minutes, then washed with DEPC-treated water. Slides were placed into a vegetable steamer at 99 °C, first into a solution of DEPC-treated water for 10 seconds, and then into a 1× Target Retrieval Reagent solution for 5 minutes. Slides were then rinsed with DEPC-water and dried for 60 minutes at RT, then left to dry overnight at RT. Diluted RNAscope protease III solution (1:1 in PBS) was added to each section and incubated for 20 minutes at 40 °C, then rinsed with DEPC-water. Prewarmed (40 °C for 10 minutes) RNA target probes against *Kiss1* transcript (Advanced Cell Diagnostics, CA, USA Cat # 500 141) were applied to one series of medial ARC sections to hybridize for 2 hours at 40 °C. After probe hybridization, amplification solutions (AMP1, AMP2, AMP3) and fluorophore Opal 690 (676/694 nm; Cat # FP1497001KT; Akoya Biosciences, MA, USA) were applied to the sections at 1:500 dilution. Signal was developed using RNAscope HRP C1, C2, C3 solutions. Nuclei were stained with DAPI and slides were cover-slipped using Prolong Gold Antifade liquid mountant (Southern Biotech, 0100-01). Images were acquired with a Keyence fluorescence microscope, using a 20× objective. An image was taken that captured both sides of the ARC; however, images were quantified using unilateral (one side) cell counts. Per animal, 2 to 3 sections were quantified and the ARC boundaries remained consistent throughout quantification. A manual cell count was conducted for positively stained cells for *Kiss1* mRNA transcripts with the assistance of the cell counter function on FIJI, in a designated boundary drawn around the ARC using the Paxinos and Franklin mouse brain atlas. The average pixel intensity for the ARC region was quantified using CellProfiler, where a mask was created outlining the ARC in each image and the average pixel intensity was defined only within the boundary outlined. The mean pixel intensities presented are the average of all pixel intensities from *Kiss1* mRNA staining within the boundaries of the ARC from that mouse.

### Liver histology

Oil-red-O staining was performed to assess lipid levels in liver samples as previously described (41). Twelve-micron-thick liver sections were sectioned at −20 °C on a cryostat and stored at −80 °C (experiment 1 and 3, n = 6-7 per group). Oil-red O (2.5 g) was dissolved in 400 mL of 99% isopropyl to make a stock solution, which was further diluted in distilled water to make a working solution. The working solution was filtered prior to use, and sections were incubated in the working solution for 5 minutes, then rinsed in cold water for 30 minutes prior to sealing with nail varnish. Sections were imaged within 24 hours of staining on an Olympus 1501 microscope (20×). The red lipid stain was quantified on ImageJ software using 5 regions of interest per animal and the investigator was blinded to treatment group. Thresholding was determined and remained consistent throughout the analysis. Staining is initially presented as a lipid to total tissue ratio, with overall lipid accumulation quantified by thresholding the stained image and measuring the area fraction (%) of Oil-red-O-positive staining relative to the analyzed image area. RGB images were converted to an RGB stack, and the second channel was selected for analysis. Automatic thresholding was applied to determine signal segmentation parameters, with a manual threshold range (0-140 intensity units) subsequently applied to define positive signal. Images were further analyzed to investigate the average lipid droplet size (µm^2^) and lipid droplet area (%Area), representing the proportion of the analyzed image occupied by lipid droplets. Images were converted to 8-bit and thresholded to generate binary masks of lipid-positive regions. Morphological opening, hole filling, and watershed segmentation were applied to improve droplet separation. Particles were quantified with a minimum size threshold of 1 µm^2^ and circularity range of 0.30 to 1.00. Area, diameter, and shape descriptors were recorded.

### Luteinizing hormone assay

At the time of euthanasia in experiments 1 and 3, whole blood was taken from the tail using heparinized capillary tubes. In experiment 1, blood was spun down and plasma was snap frozen on dry ice and prior to the assay, 4 μL of plasma was diluted in 110 mL of PBS with 0.05% Tween-20. In experiment 3, a quantity of 4 μL of whole blood was diluted in 50 μL PBS with 0.05% Tween-20 and snap frozen on dry ice. Samples were stored at −20 °C until LH was measured by an ultrasensitive sandwich enzyme-linked immunosorbent assay (ELISA), as previously described and modified recently (42, 43). Briefly, 96-well high-affinity binding plates were coated with 50 μL per well of mouse monoclonal anti-bovine LH 518B7 antibody (dilution 1:1000 in PBS; UC Davis, catalog No. 518B7, lot No. 13; RRID: AB_2665514). A commercially available mouse monoclonal LH 5303 SPRN-5 antibody was biotinylated (dilution 1:1000, Medix Biochemica, catalog No. 100588; lot No. 0046906; RRID: AB_2784503) and used to detect bound LH in conjunction with a polyclonal-horseradish peroxidase conjugate streptavidin (Thermo Fisher, catalog No. N200, dilution 1:8000). The LH concentration was determined using a mouse LH-RP reference provided by Albert F. Parlow (National Hormone and Pituitary Program, Torrance, CA, USA). Within the 17βE2 treatment groups, 5 animals in experiment 1 and 7 animals in experiment 3 had undetectable LH levels, so the lowest value detected in either assay was recorded. The intra-assay coefficient was 1.8% and the inter-assay coefficient was 0.52%.

### Statistical analyses

Statistics were performed using GraphPad Prism software (version 10). For each measured parameter, either an ordinary one-way analysis of variance (ANOVA) or Kruskal Wallis test was performed. A one-way ANOVA was used if the data were normally distributed and variance was similar between groups, but if these assumptions were not met, a Kruskal Wall test was applied. For body weight, food intake, and glucose levels across the glucose tolerance test, a 2-way repeated measures ANOVA was performed, including body weight change from baseline, average daily food intake, or glucose levels as the repeated dependent variable and treatment as an independent variable. Where a main group effect was observed, a Fisher's LSD post hoc test (or a Dunn's post hoc test for nonparametric comparisons) was performed to determine whether 17βE2 or DHED treatment groups differed from controls. Results are presented as mean ± SEM, unless otherwise stated.

## Results

### Experiment 1

#### Effects of DHED administration on ovary weight, LH concentration, and progesterone receptor expression

A previous study has shown that DHED treatment can provide benefits when provided at the same dose as 17βE2 in rodent models investigating depressive-like behavior and working memory, without influencing uterine weight (33). We therefore first tested DHED treatment at the same dose as 17βE2, with each hormone administered at 5 μg/day. After 25 days of treatment, 17βE2-treated animals had significantly increased uterine weights in comparison with vehicle-treated animals, (Fig. 1b, *P* < 0.0001 overall effect of treatment, *P* < 0.0001 for Dunn's post hoc comparison between 17βE2-treated and control animals). DHED-treated animals showed no significant increase in uterine weight compared to controls and had lighter uterine weights than 17βE2-treated animals (Fig. 1b, *P* = 0.013, comparison between 17βE2- and DHED-treated animals).

Plasma LH levels were significantly reduced to almost completely undetectable levels in response to 17βE2 treatment in OVX animals (Fig. 1c, *P* < 0.0001 overall effect of treatment, *P* < 0.001 for Dunn's post hoc comparison between 17βE2-treated and control animals), indicative of the strong negative feedback effects of 17βE2 at the level of the hypothalamus. DHED, when administered at the same dose of 5 μg/day, failed to significantly change plasma LH levels relative to controls.

PR within the ARC was significantly increased in response to 17βE2 treatment (Fig. 1d and 1e, *P* < 0.0001 overall effect of treatment, *P* < 0.0001 for Dunn's post hoc comparison between 17βE2-treated and control animals). While substantial PR abundance was detected in 5 of 7 animals following DHED treatment, the averaged observed increase did not reach significance compared to controls (Fig. 1d and 1e, *P* = 0.0735) and was significantly different to the observed increase with 17βE2 (Fig. 1d and 1e, *P* = 0.0210).

#### 17βE2, but not DHED treatment, significantly reduces body weight and food intake in OVX mice exposed to a HFD

Within 6 days after the initiation of 17βE2 treatment, mice began to significantly reduce their body weight (Fig. 2a, *P* = 0.0003 overall effect of treatment across the measuring period, *P* < 0.05 effect of 17βE2 in comparison to control animals at each time point after 6 days). DHED administration did not recapitulate this protective effect of reduced body weight, with DHED-treated individuals showing no significant difference in body weight compared to controls. Food intake was also significantly reduced almost immediately after the initiation of treatment in the 17βE2 group (Fig. 2b, *P* < 0.0001 overall effect of treatment, *P* < 0.05 effect of 17βE2 relative to controls at denoted time points), but DHED did not influence food intake compared to controls.

**Figure 2:**
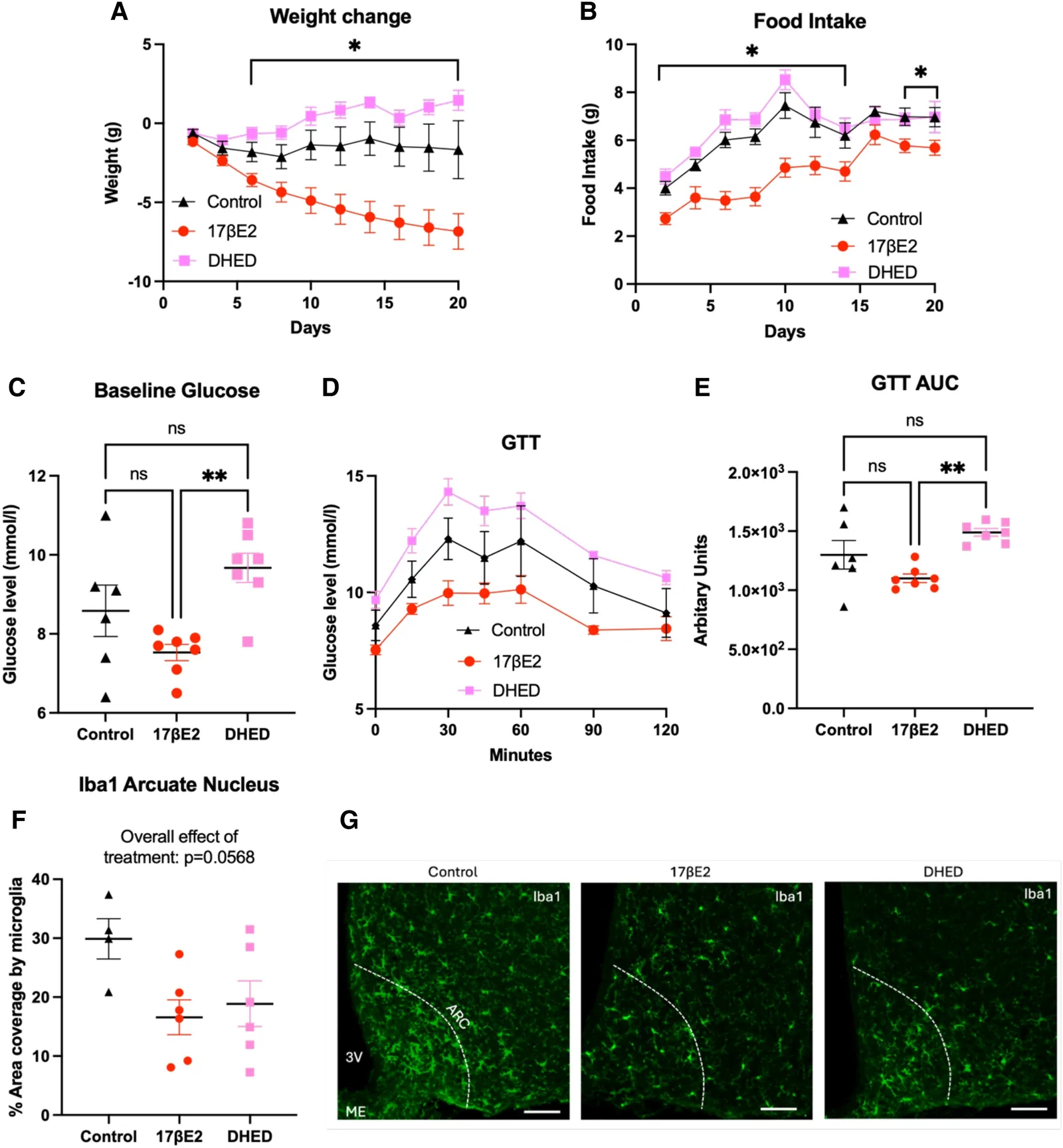
17βE2, but not DHED treatment, significantly improves metabolic parameters. a, Body weight change after initiation of hormone treatment (17βE2 or DHED) following 8 weeks on HFD. b, Food consumption (per each mouse for 2 days). c, Baseline glucose levels. d, Glucose tolerance. e, area under the curve. a, b: *P < 0.05 effect of 17βE2 relative to controls at denoted time points (Fisher's LSD post hoc test). c, e: **P < 0.01 (Dunn's post hoc test). f, Percent area covered by microglia within the ARC. g, Representative images demonstrating Iba1 (microglia) within the ARC following vehicle control, 17βE2, or DHED treatment. Scale bar = 66 µm. n = 6-7 per group. Error bars show mean ± SEM. Abbreviations: ARC, arcuate nucleus; 3V, third ventricle; ME, median eminence.

#### Glucose tolerance is not improved in response to either DHED or 17βE2 treatment

In contrast to the hormone's effect on body weight and food intake, 17βE2 treatment did not significantly lower glucose levels compared to control animals at the baseline (Fig. 2c, *P* = 0.0092 overall effect of treatment, *P* = 0.2131 effect of 17βE2 relative to controls). There was a trend across the experiment for significantly improved glucose tolerance in 17βE2-treated mice, most notably 30 minutes after glucose injection (*P* = 0.0535, effect of 17βE2 relative to controls); however, no significant difference was observed between groups at any timepoint after glucose injection (Fig. 2d). While baseline glucose levels and area under the curve (AUC) showed an overall effect of treatment (Fig. 2c and 2e, *P* < 0.05) for each, the post hoc analysis revealed this was likely driven by differences between hormone-treated animals (*P* < 0.01, effect of 17βE2 in comparison with DHED-treated animals), as there were no differences between either 17βE2- or DHED-treated animals in comparison with controls for any parameter. Taken together, these results illustrate that neither 17βE2 nor DHED improves glucose control in these mice.

We next assessed whether DHED administration could recapitulate central anti-inflammatory actions associated with estrogen treatment. The overall difference between groups for the percentage area covered by microglia in the ARC failed to reach significance (Fig. 2f and 2g  *P* = 0.0568 overall effect of treatment). However, we cautiously note that both 17βE2 and DHED animals tended to have lower levels than controls, justifying further investigation in later experiments (see experiment 3).

#### Liver adiposity is not reduced in response to either 17βE2 or DHED treatment

17βE2 has previously been reported to reduce liver adiposity in OVX female rodents (44); however, we did not observe a reduction in hepatic lipid levels in 17βE2-treated mice (Fig. 3a and 3d, *P* = 0.2550 effect of 17βE2 in comparison to control animals). While we observed an overall effect of treatment (Fig. 3a, *P* = 0.0492), this is not explained by any comparison to control animals and rather is driven by differences between hormone-treated groups (*P* = 0.0492 overall effect of treatment, *P* = 0.0162 comparison between 17βE2 and DHED animals). We also observed no change in lipid abundance in DHED-treated mice compared to controls (Fig. 3a). Additionally, we quantified the average lipid droplet size and distribution throughout the liver and found no differences between either 17βE2 or DHED in comparison to controls for these parameters (*P* = 0.0506, *P* = 0.0810 overall effect of treatment in Fig. 3b and 3c, respectively).

**Figure 3:**
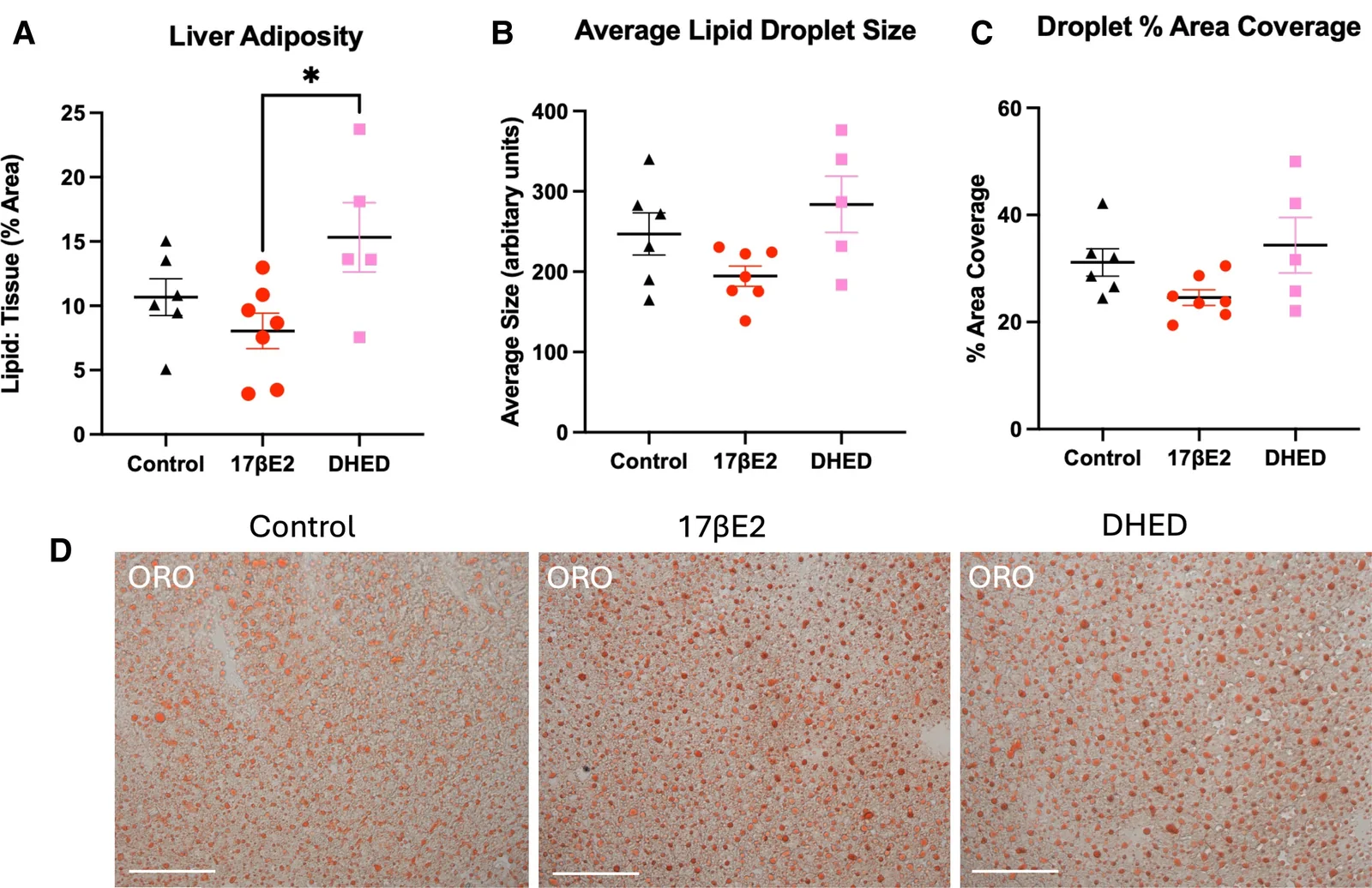
17βE2 or DHED treatment does not significantly alter hepatic adiposity. a, Overall liver lipid levels. b, Average lipid droplet size. c, Percentage area covered by lipid droplets. d, Representative images showing liver lipid levels in response to treatment following Oil Red O (ORO) staining. *P < 0.05, (Dunn's post hoc test). n = 6-7 per group. Error bars show mean ± SEM. Scale bar = 75 µm.

### Experiment 2

#### DHED increases progesterone receptor expression in the ARC in a dose dependent manner when delivered over 48 hours

We subsequently performed an acute study over 48 hours to ascertain the dose of DHED required to elicit similar hypothalamic responses as 17βE2. Two days of 17βE2 treatment resulted in significantly heavier uterine weights than control animals (Fig. 4b, *P* < 0.01 effect of 17βE2). In contrast, none of the DHED doses influenced uterine weight compared to control animals (Fig. 4b, overall effect of treatment *P* < 0.05, effect of DHED treatment at any dose compared to controls *P* > 0.05).

**Figure 4:**
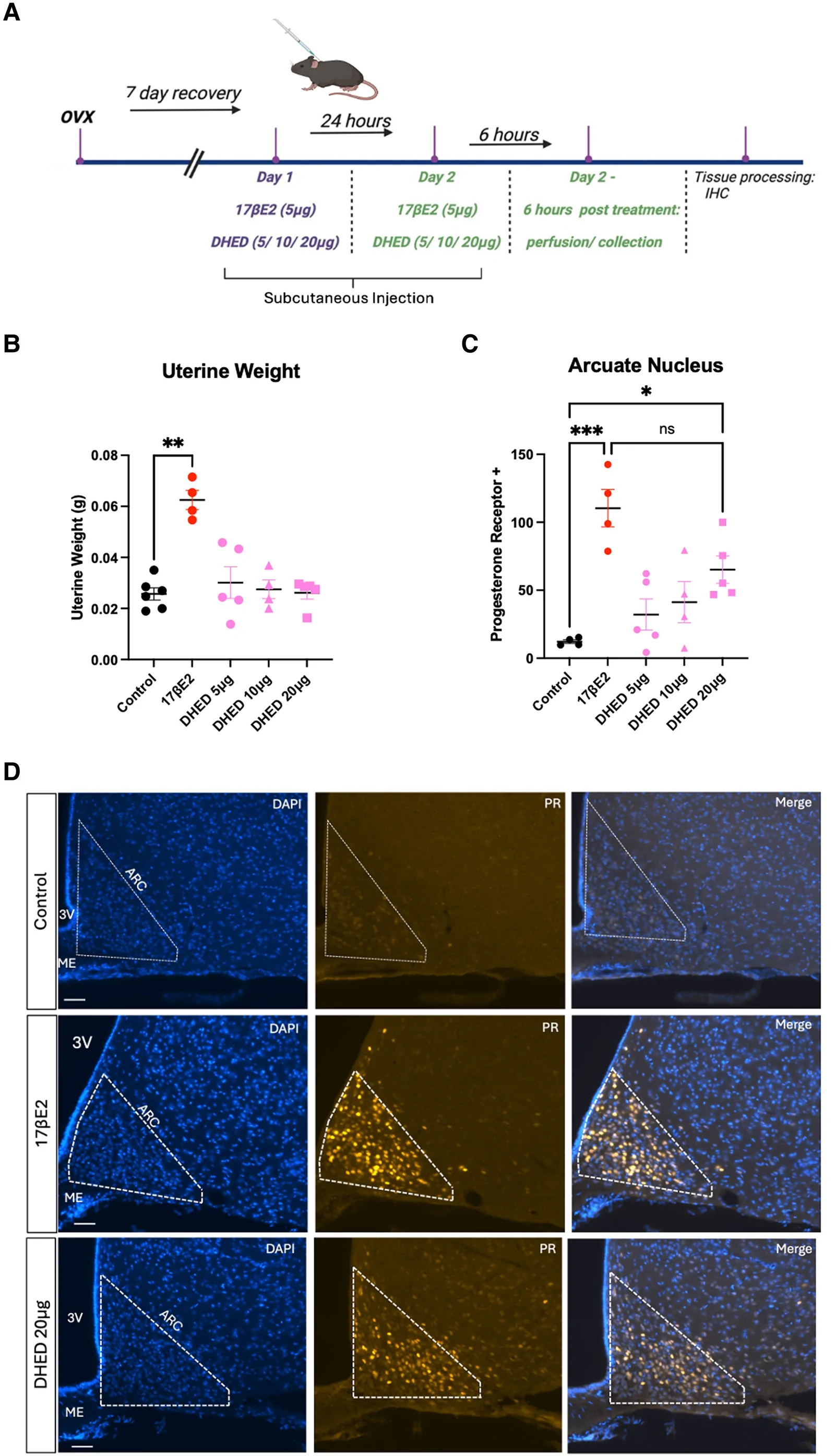
Experiment 2. a, timeline: Mice were ovariectomized, allowed to recover, then received either vehicle (n = 6), 17βE2 (5 μg/day, n = 4), or DHED (5 μg/day [n = 5], 10 μg/day [n = 4] or 20 μg/day [n = 5]) once daily for 2 days via subcutaneous injection. Six hours post treatment on day 2, animals were euthanized and perfused with 4% PFA. b, Uterine weight after 48 hours of hormone treatment. c, PR abundance in the ARC. d, Representative images of PR immunoreactivity (orange) expression in the ARC (DAPI, PR only and merged channels) following control, 17βE2, or DHED 20 µg (see Figure S1 for DHED 5 µg and 10 µg images) (45). *P < 0.05, **P < 0.01, ***P < 0.001 (Dunn's post hoc test). Error bars show mean ± SEM. Scale bar = 50 µm. Abbreviations: ARC, arcuate nucleus; 3V, third ventricle; ME, median eminence.

When administered acutely at the same dose of 5 µg as used in the previous experiment, 17βE2 significantly increased PR expression after 48 hours (Fig. 4c, *P* < 0.05 overall treatment effect, ***P < 0.001 effect of 17βE2). However, 48 hours of DHED treatment at the same dosage as 17βE2 or administered at a two-fold higher dose of 10 µg/day failed to significantly increase PR expression in this acute treatment setting (*P* > 0.05). A significant increase in the number of cells expressing PR was only observed when DHED was administered at a 4-fold increase of 20 µg/day (Fig. 4c and 4d, *P* = 0.026 effect of 20 µg/day DHED treatment relative to controls). There was also no statistical difference observed in number of PR+ cells in the ARC between 17βE2 and DHED administered at 20 µg/day (see Supplementary Figure 1 (45) for DHED 5 µg and 10 µg images).

#### Acute administration of 17βE2 for 48 hours reduces *Kiss1* mRNA staining intensity within the ARC, with DHED showing a similar response

Following acute subcutaneous administration of either 17βE2 or DHED (20 µg/day only), the number of cells expressing individual *Kiss1* mRNA transcripts within the ARC were quantified via RNAscope. Neither 17βE2 nor DHED treatment significantly influenced the number of positively stained cells for *Kiss1* mRNA (overall effect of treatment *P* = 0.3559, Fig. 5a). However, when the overall pixel intensity of *Kiss1* mRNA staining within the ARC was quantified using CellProfiler, the 17βE2-treated group demonstrated a significant decrease in *Kiss1* mRNA expression (overall effect of treatment *P* = 0.0009, effect of 17βE2 *P* = 0.0027, Fig. 5b and 5c) as previously reported (44). DHED treatment at 20 µg showed a similar reduction compared to controls although this just failed to reach statistical significance (*P* = 0.0509).

**Figure 5:**
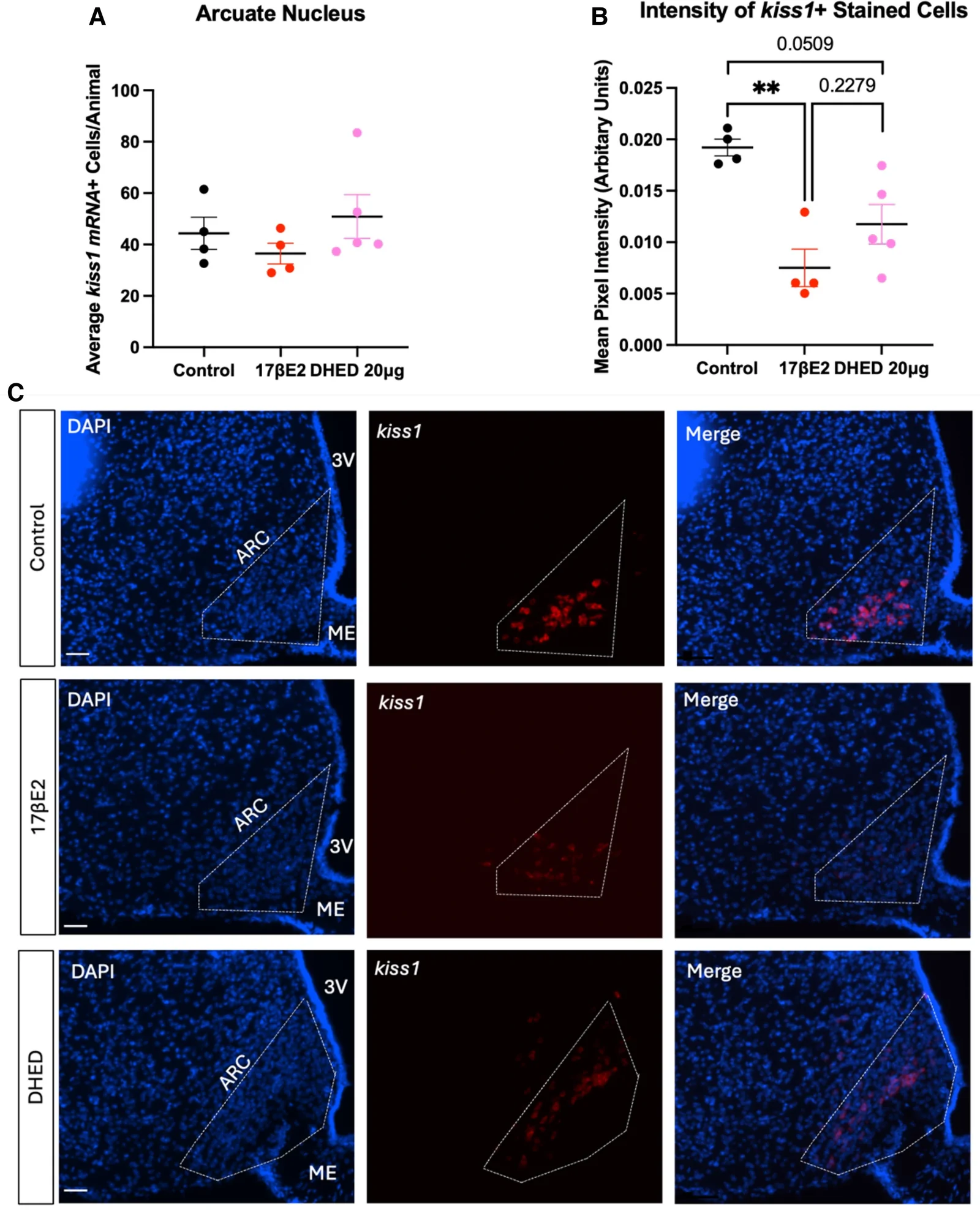
17βE2 significantly reduces the intensity of positively stained cells expressing *Kiss1* within the ARC, while DHED treatment strongly trends toward this reduction. a, Number of cells within the ARC expressing *Kiss1* mRNA. b, Mean pixel intensity of cells expressing *Kiss1* mRNA within the ARC. c, Representative images showing *Kiss1* staining within the ARC following vehicle control, 17βE2, or DHED (20 μg) treatment. **P < 0.01 (Dunn's post hoc test). Scale bar = 50 µm. Error bars show mean ± SEM. n = 4-5 per group. Each dot represents the average number of positively stained *Kiss1* cells for one mouse in a, or the average pixel intensity of positively stained *Kiss1* cells for one mouse in b. Abbreviations: ARC, arcuate nucleus; 3V, third ventricle; ME, median eminence. n = 4-5 per group.

### Experiment 3

#### DHED increases PR expression and reduces neuroinflammation in the ARC but does not influence metabolic health at any dose

Given that a higher dose of acutely administered DHED (20 µg) was required to see estradiol-like actions in the brain, we carried out a longer-term study with a range of increasing DHED doses. After 14 days of 17βE2 treatment, animals had significantly increased uterine weights in comparison with vehicle-treated animals (Fig. 6b, *P* < 0.0001, overall effect of treatment). DHED at 5 µg/day had no significant effect on uterine weight compared to controls but showed a pattern that was reflective of that seen in the first experiment (Fig. 6b, *P* = 0.0963). Animals treated with a higher dose of DHED at either 10 or 20 µg/day had increased uterine weights relative to controls (*P* = 0.0042, *P* = 0.0099 effect of 10 or 20 µg/day respectively relative to controls), but the 20 µg/day group were significantly lighter than 17βE2-treated animals (*P* < 0.05).

**Figure 6:**
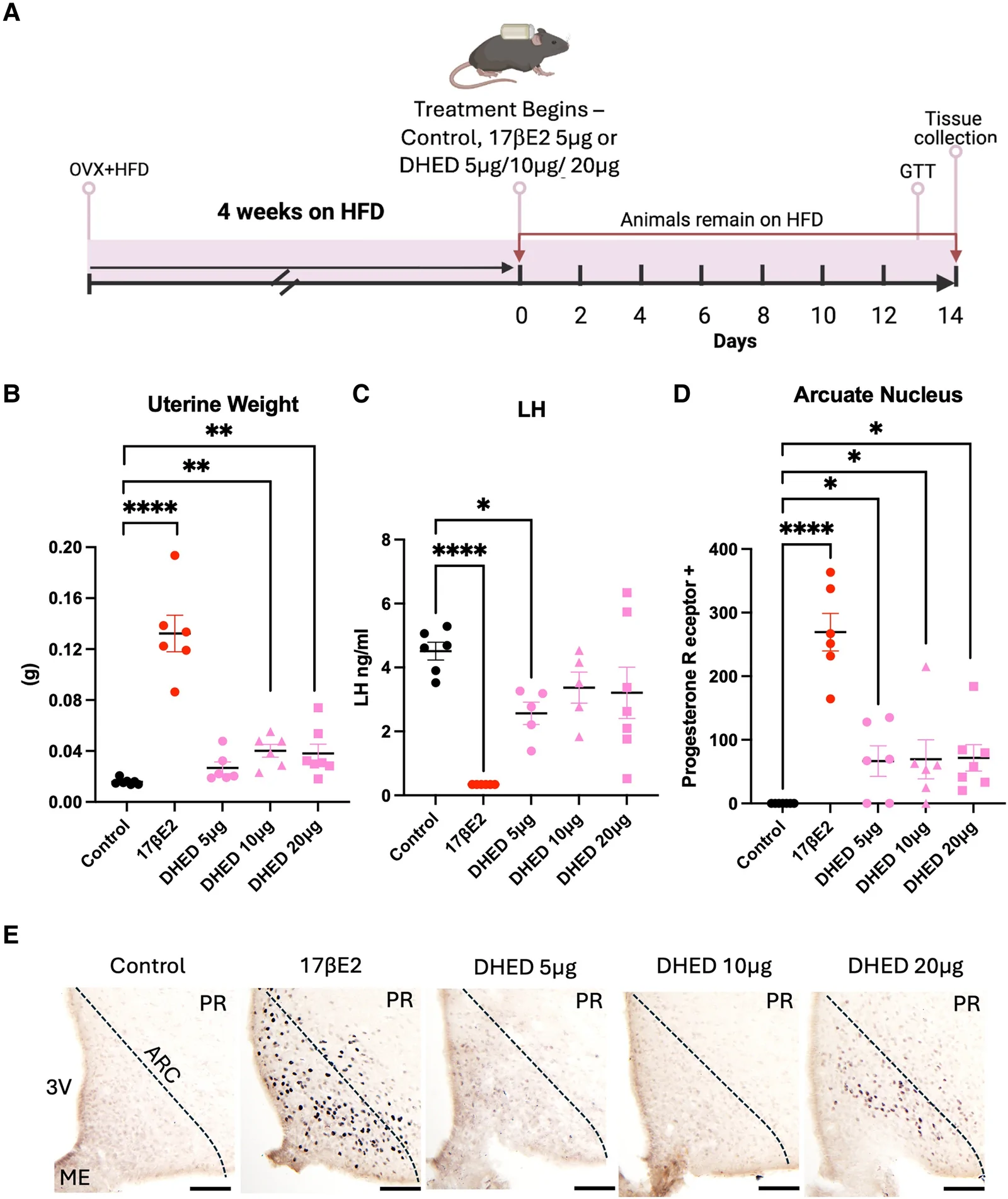
Experiment 3: DHED increases PR expression in the ARC. a, Timeline. Animals were ovariectomized and placed on a 45% HFD for 4 weeks. Mice underwent a second surgery to insert osmotic mini-pumps to deliver treatment: vehicle (n = 7), 17βE2 (5 μg/day, n = 6), or DHED (5 μg/day [n = 6], 10 μg/day [n = 6], 20 μg/day [n = 7]) for 14 days. At the end of the experiment all mice were perfused and tissues collected. b, Uterine weight in response to 17βE2 or increasing doses of DHED. c, LH levels. d, PR-positive cells in the ARC. e, Representative images showing PR expression in the ARC following control, 17βE2, DHED 5 µg, DHED 10 µg, or DHED 20 µg. *P < 0.05, **P < 0.01 ****P < 0.0001 (Dunn's post hoc test). Error bars show mean ± SEM. Scale bar = 50 µm. Abbreviations: ARC, arcuate nucleus; 3V, third ventricle; ME, median eminence.

Similar to experiment 1, 17βE2 treatment was extremely effective in reducing LH levels to undetectable levels in OVX animals (Fig. 6c, *P* < 0.001 overall effect of treatment, *P* < 0.0001 effect of 17βE2 relative to control animals). All DHED-treated animals showed a similar pattern in their LH levels, although only the group that received 5 µg/day showed a statistically significant reduction in circulating LH levels compared to controls (*P* < 0.05 effect of DHED 5 µg relative to controls). For the 10 or 20 µg/day DHED groups, there was no significant difference in LH levels compared to controls (*P* = 0.2463, *P* = 0.1205, effect of 10 or 20 µg/day, respectively, relative to controls).

The number of PR-expressing cells was robustly increased in response to chronic 17βE2 treatment (Fig. 6d and 6e, *P* < 0.001 overall effect of treatment, *P* < 0.0001 effect of 17βE2 relative to control animals). Chronic DHED treatment also increased PR expression at all of the 3 doses tested (Fig. 6d and 6e, *P* < 0.05 effect of each dose in comparison to control animals), although to a lower degree.

#### Increasing DHED dosages does not improve body weight or food intake

Over the first week of 17βE2 treatment, mice began to reduce their body weight (Fig. 7a), which was consistent with our previous experiment (*P* < 0.001 overall effect of treatment across the measuring period, see supplementary Figure S2 (45) for absolute body weight change over both experiments). DHED treatment did not recapitulate this protective effect of reduced body weight at any dose, with DHED-treated individuals showing no significant difference in body weight compared to controls. Food intake was also significantly reduced almost immediately after the initiation of treatment in the 17βE2 group (Fig. 7b, *P* < 0.01 overall effect of treatment across the measuring period) but none of the doses of DHED (5, 10, 20 µg) significantly influenced food intake in this cohort.

**Figure 7:**
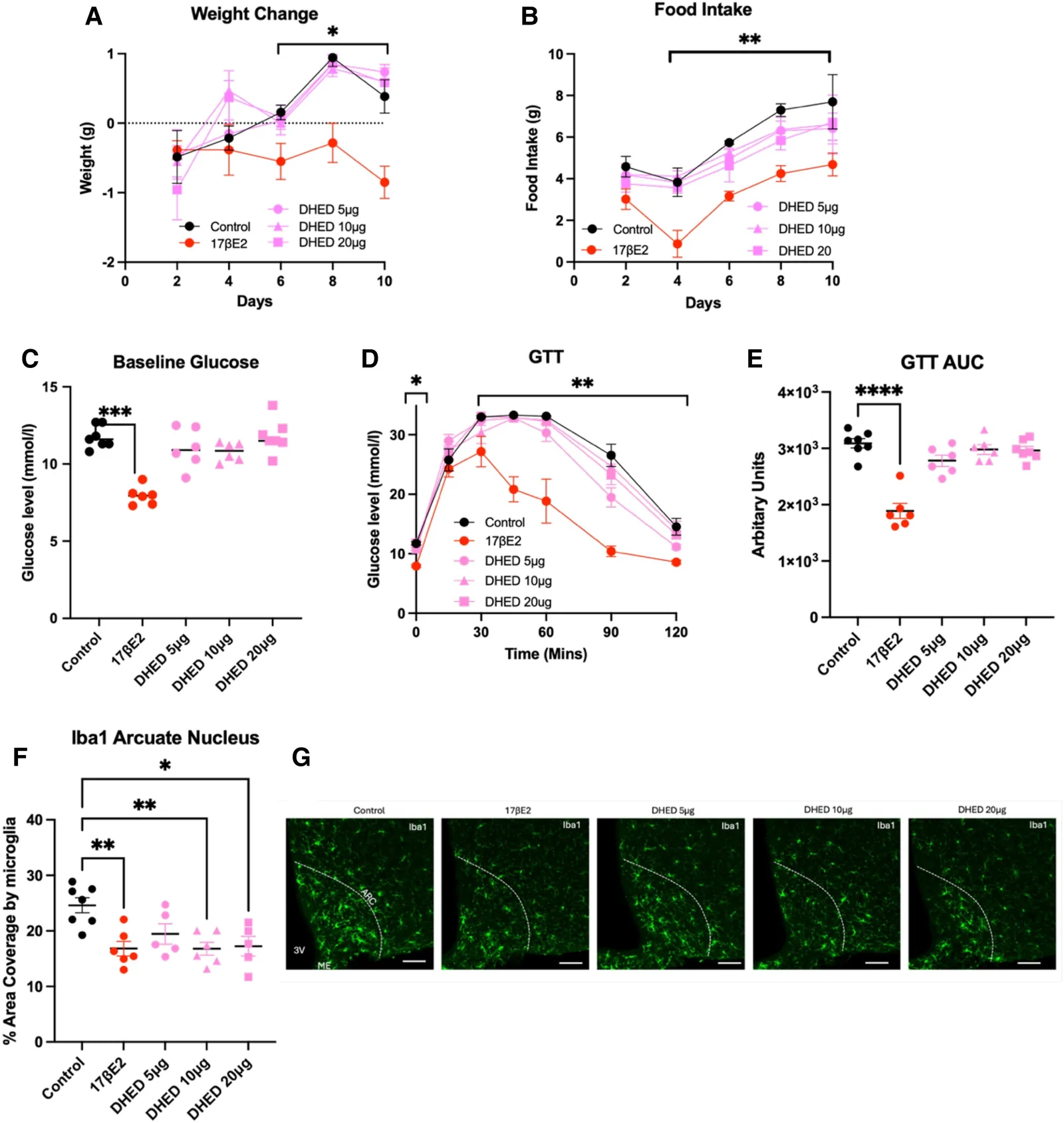
Increasing doses of DHED does not improve metabolic health in OVX obese mice, yet it significantly reduces microglial percentage area coverage in the ARC. a, Body weight change in response to 17βE2 or DHED treatment. b, Food intake (per each mouse for 2 days). c, Baseline glucose. d, Glucose tolerance. e, Area under the curve. f, Percent area covered by microglia within the ARC. g, Representative images demonstrating Iba1 (microglia) within the ARC following vehicle control, 17βE2, or DHED 5, 10, or 20 µg/day treatment. a, b, d, *P < 0.05, **P < 0.01, effect of 17βE2 relative to controls from Fisher's LSD post hoc test at each timepoint. c, e, f *P < 0.05, **P < 0.01 ***P < 0.001, ****P < 0.0001 (Dunn's post hoc test). Scale bar = 66 µm. Error bars show mean ± SEM. n = 6-7 per group. Abbreviations: ARC, arcuate nucleus; 3V, third ventricle; ME, median eminence.

#### DHED treatment does not improve glucose tolerance or liver adiposity at increasing doses but does reduce microglial coverage within the ARC

17βE2 significantly improved glucose tolerance compared to controls, with animals in this group showing a reduction in baseline glucose levels (Fig. 7c, *P* = 0.0011 overall effect of treatment), reduced glucose concentration at every time point from 30 minutes post glucose injection (Fig. 7d, *P* < 0.0001 overall effect of treatment), and a significantly smaller AUC (Fig. 7e, *P* < 0.01 overall effect of treatment). In contrast, DHED treatment at either 5, 10, or 20 µg failed to have any effect on fasting glucose levels, glucose tolerance, or AUC (Fig. 7c-7e).

We next assessed the percentage area covered by microglia within the ARC as a marker of neuroinflammation. Similarly to experiment 1, 17βE2 significantly reduced the percentage area covered by microglia in the ARC (Fig. 7f and 7g, *P* < 0.05 overall effect of treatment, *P* < 0.001 effect of 17βE2 treatment relative to controls). While DHED treatment at the same dose as 17βE2 failed to induce a similar response (Fig. 7f and 7g, *P* = 0.0994 effect of DHED 5 µg relative to controls), at higher doses DHED significantly reduced the percentage area covered by microglia in the ARC (Fig. 7f and 7g, *P* < 0.01, effect of DHED 10 or 20 µg respectively relative to controls).

Liver adiposity was not significantly influenced by either 17βE2 or any dose of DHED treatment compared to controls. Similar to the first experiment, no differences in hepatic lipid abundance, average lipid droplet size or percentage coverage of lipid were observed between treatment groups (Fig. 8a and 8d, overall effect of treatment *P* = 0.2780 (a), *P* = 0.1805 (b), and *P* = 0.3987 (c).

**Figure 8:**
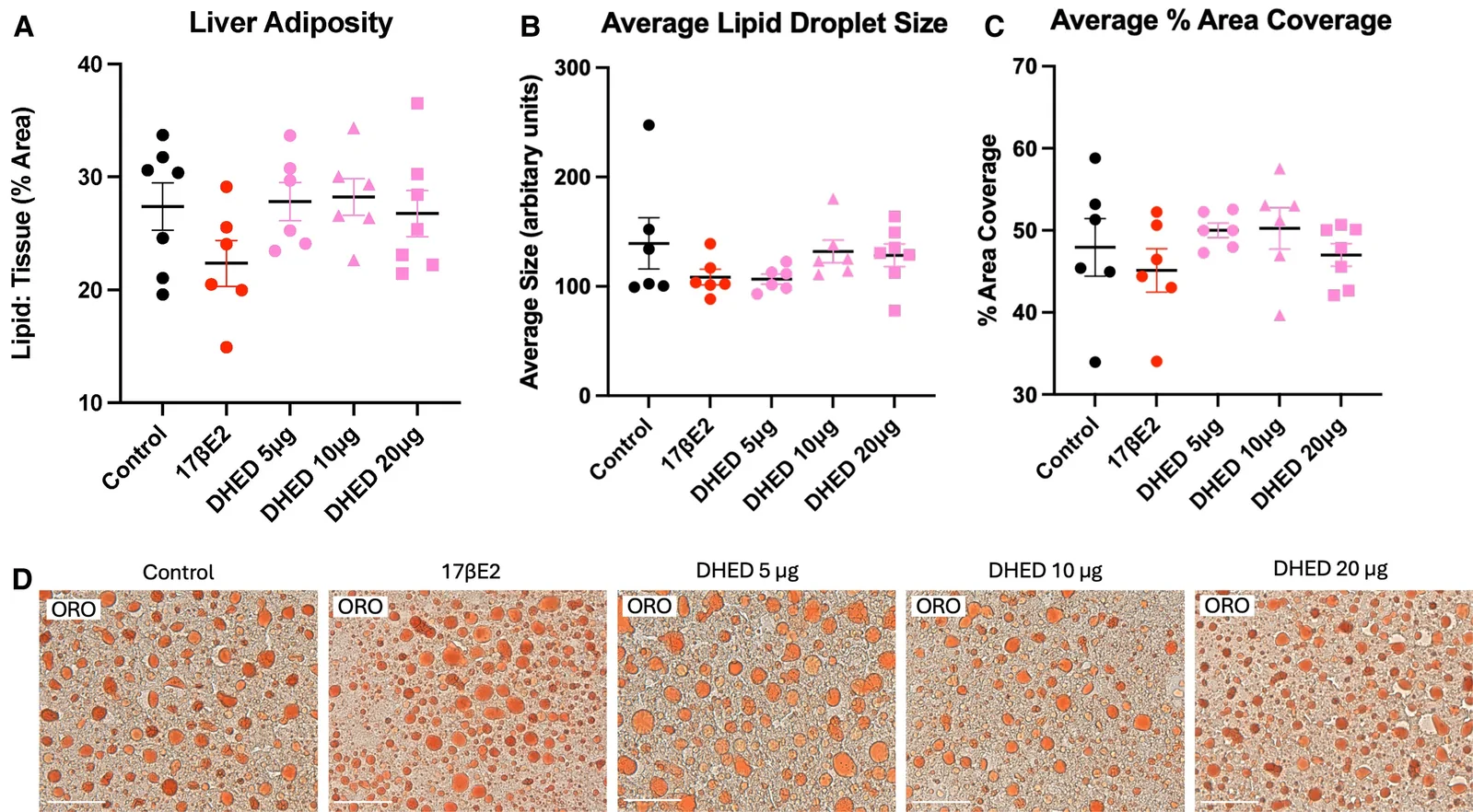
Liver adiposity is not significantly altered following treatment with 17βE2 or DHED at increasing doses. a, Overall liver lipid levels. b, Average lipid droplet size. c, Percentage area covered by lipid droplets. d, Representative images showing liver lipid levels in response to treatment following Oil Red O (ORO) staining. n = 6-7 per group. Error bars show mean ± SEM. Scale bar = 75 µm.

## Discussion

This study sought to determine whether administration of DHED, a brain-targeting prodrug of 17βE2, could recapitulate the protective metabolic effects of 17βE2 treatment in OVX mice perturbed by a HFD. Confirming previous reports, we found that 17βE2 significantly improved metabolic parameters in obese OVX female mice receiving HFD treatment (16, 17, 36, 46). In contrast, DHED administration failed to improve body weight and associated metabolic parameters at any dose tested, similar to a recent report in aged mice on a standard rodent chow diet (47). Taken together, our results suggest that exclusive targeting of the brain with 17βE2 via DHED treatment, at the same or up to 4-fold higher doses, is insufficient to improve metabolic health in OVX mice. Our study focused on body weight, food intake, and glucose tolerance as the markers of metabolic function, as these traits that are highly responsive to estrogen treatment in mice. We cannot discount the possibility that DHED treatment may have impacted other metabolic traits that were not assessed in this study, such as depot-specific changes in adipose tissue size, structure or function, or changes in a specific aspect of energy expenditure, which may not be reflected by total body weight.

The dose of 17βE2 applied here was equivalent to that used in previous studies that have shown beneficial effects of 17βE2 on metabolic health (16, 17, 36, 46). Previous studies investigating DHED's capacity to provide benefits in cognitive decline, hot flush, and depression have suggested that the same dose of the prodrug as 17βE2 is sufficient to alleviate symptoms (30, 31, 33). Across our study, direct administration of 17βE2 significantly increased uterine weight compared to controls, indicating the presence of this hormone in the circulation. In contrast, short-term or low-dose chronic DHED administration had no effect on uterine weight, yet higher doses of DHED delivered over 14 days resulted in an approximately 2-fold increase in uterine weight relative to controls. However, this increase was modest when compared with the proliferative effects of 17βE2, with animals in this treatment group showing an approximately 6-fold increase in uterine weight. Given that no previous study has reported an increase on uterine weight and previous extensive characterization has failed to detect DHED-derived 17βE2 in peripheral tissues (30, 31, 47), it is possible that perturbation by a HFD may partially explain this increase in uterine weight.

HFD induces chronic inflammation and an increase in oxidative stress in the brain, including within the ARC (48, 49). Resulting neuroinflammation can lead to a wide array of pathophysiological effects in the brain, including mitochondrial dysfunction and neuronal death (50). Given that neuroinflammatory populations can also alter blood brain barrier permeability, it is plausible that a HFD is altering typical brain signaling actions and is causing some leakage of DHED from the brain back into the circulation, particularly at high concentrations (51), which could explain the small effects of DHED treatment on uterus weight. It is also possible that a HFD may be altering the enzymatic conversion of DHED into 17βE2 within the ARC, which may explain the limited increase in PR expression abundance and change in *Kiss1* mRNA expression we observed after treatment with the prodrug. Unfortunately, it was not possible to measure 17βE2 in this study, with most commercially available ELISAs lacking sufficient specificity. Future studies are required to determine whether DHED's conversion to 17βE2 is impacted by diet or varies across brain regions that control body weight. These studies should utilize liquid chromatography–tandem mass spectrometry (LC-MS/MS)-based bioassays (30, 31) to measure 17βE2 in both the circulation and distinct brain regions, including the hypothalamus, under normal chow and HFD conditions.

The lack of metabolic response to DHED treatment could be the result of 2 main non-mutually exclusive reasons: either that DHED treatment fails to replicate the central signaling effects of 17βE2 within hypothalamic regions that control body weight and metabolism, or that peripheral estrogen signaling is also required for metabolic protection. Regarding the first reason, we used PR expression as a reporter for estrogen receptor actions within the hypothalamus in all 3 experiments as a proxy to assess estradiol circulation within the hypothalamus (52-54). Previously, the only brain nucleus that had been probed for PR expression following DHED treatment was the preoptic area (POA), where DHED was shown to increase expression to the same degree as 17βE2 in OVX rats when delivered at the same dose (30, 31). By contrast, in our study, DHED failed to robustly increase PR expression in the ARC, with consistent increases only detected when DHED was delivered at a 4-fold higher dose to 17βE2. We also observed that DHED did not reduce circulating LH levels to the same degree as 17βE2 treatment. In mice, the reduction in LH levels with estrogen treatment in OVX mice is largely linked to hypothalamic signaling actions on kisspeptin neurons (55). While we observed that short-term treatment with DHED could induce some changes in kisspeptin expression in our second study, it is likely that stimulation with DHED treatment was insufficient to substantially induce negative feedback on the hypothalamus-pituitary-gonadal axis and reduce LH levels. This supports the argument that DHED treatment, at least at the doses used in this study, fails to fully replicate the signaling actions of 17βE2 in the hypothalamus.

Regarding the second idea, that peripheral actions of estrogens are required for metabolic protection, it is plausible that integrated signals between central estrogen actions and downstream tissues are required to drive protective metabolic effects. Ablation of peripheral ERs disrupts metabolic health, by altering triglyceride levels (liver-specific knockout models), enhancing adipocyte mass (adipocyte-specific knockouts) and disrupting bone turnover (global knockouts) (56-59). Because DHED is inactive (or has very limited actions) outside of the brain, these peripheral effects of 17βE2 would be absent in DHED-treated animals. This may explain why the metabolic improvements we observed with 17βE2 were absent in DHED-treated animals, and why others have shown cognitive improvements with DHED treatment but a lack of metabolic protection (47).

It is interesting, however, that despite failing to improve direct metabolic parameters like body weight, food intake, and liver adiposity, DHED treatment reduced microglial coverage within the ARC. Pro-inflammatory microglia increase with obesity and metabolic dysfunction and contribute to increased weight gain and metabolic dysfunction (60, 61). It is well established that 17βE2 can reduce microgliosis in OVX mice (49, 62), although the mechanisms, receptors, and cell locations that drive this effect are still under debate. As stated above, it is possible that DHED conversion to 17βE2 may be insufficient within the hypothalamus to fully engage all cellular pathways required to reduce body weight and food intake. However, the reduction of microgliosis within the ARC suggests that DHED treatment is capable of protecting against some negative effects of metabolic dysfunction and HFD feeding. This could conceivably occur if a different level/cellular location of estrogen signaling is required to drive changes in microglia abundance compared to that required to act on neuronal populations that directly control food intake (63). Regardless, the effects of DHED on microglia and hypothalamic inflammation are worthy of further investigation, given the strong links between neuroinflammation and a variety of metabolic and age-related diseases (26, 64-66).

To conclude, our investigations reveal that treatment with the DHED prodrug failed to offer any benefit on metabolic health in obese, OVX mice, despite evidence that this treatment is inducing at least some aspects of estrogen signaling within the ARC of the hypothalamus. While further analysis of DHED's actions within the hypothalamus and in the context of a HFD are required, future directions focusing on alternatives to traditional MHT could benefit from integrated approaches between 17βE2's actions in the brain and periphery.

## Data Availability

The data that support the findings of this study are available upon reasonable request from the corresponding author. Data for supplementary figures are available at https://doi.org/10.6084/m9.figshare.32095639.

## Abbreviations

17βE2: 17 beta-estradiol
ANOVA: analysis of variance
ARC: arcuate nucleus
AUC: area under the curve
ELISA: enzyme-linked immunosorbent assay
Erα: estrogen receptor alpha
HFD: high fat diet
LH: luteinizing hormone
MHT: menopausal hormone therapy
NGS: normal goat serum
OCT: optimal cutting temperature compound
OVX: ovariectomized
PBS: phosphate-buffered saline
PFA: paraformaldehyde
PR: progesterone receptor
RT: room temperature
